# Novel Bioceramic Urethral Bulking Agents Elicit Improved Host Tissue Responses in a Rat Model

**DOI:** 10.1155/2016/1282531

**Published:** 2016-08-29

**Authors:** Travis K. Mann-Gow, Benjamin J. King, Ahmed El-Ghannam, Christine Knabe-Ducheyne, Masatoshi Kida, Ole M. Dall, Jan Krhut, Peter Zvara

**Affiliations:** ^1^Department of Surgery, University of Vermont, Burlington, VT 05405, USA; ^2^Department of Mechanical Engineering and Engineering Science, University of North Carolina at Charlotte, Charlotte, NC 28223, USA; ^3^Department of Medicine, Philipps University of Marburg, 35039 Marburg, Germany; ^4^Department of Pathology and Laboratory Medicine, University of Vermont, Burlington, VT 05405, USA; ^5^TAK Biopharma, 2000 Frederiksberg, Denmark; ^6^Department of Surgical Sciences, Ostrava University, 703 00 Ostrava, Czech Republic; ^7^Department of Urology and Biomedical Laboratory, Clinical Institute, University of Southern Denmark, 5000 Odense, Denmark

## Abstract

*Objectives*. To test the physical properties and host response to the bioceramic particles, silica-calcium phosphate (SCPC10) and Cristobalite, in a rat animal model and compare their biocompatibility to the current clinically utilized urethral bulking materials.* Material and Methods*. The novel bulking materials, SCPC10 and Cristobalite, were suspended in hyaluronic acid sodium salt and injected into the mid urethra of a rat. Additional animals were injected with bulking materials currently in clinical use. Physiological response was assessed using voiding trials, and host tissue response was evaluated using hard tissue histology and immunohistochemical analysis. Distant organs were evaluated for the presence of particles or their components.* Results*. Histological analysis of the urethral tissue five months after injection showed that both SCPC10 and Cristobalite induced a more robust fibroblastic and histiocytic reaction, promoting integration and encapsulation of the particle aggregates, leading to a larger bulking effect. Concentrations of Ca, Na, Si, and P ions in the experimental groups were comparable to control animals.* Conclusions*. This side-by-side examination of urethral bulking agents using a rat animal model and hard tissue histology techniques compared two newly developed bioactive ceramic particles to three of the currently used bulking agents. The local host tissue response and bulking effects of bioceramic particles were superior while also possessing a comparable safety profile.

## 1. Introduction

Approximately 130,000 surgical procedures are performed annually to treat stress urinary incontinence (SUI) in the US [1]. Urethral bulking therapy (UBT) was used frequently in the 1990s but lost popularity due to three main reasons: first, disappointing long-term efficacy; second, complications related to local host tissue response and distant tissue migration; and third, a dramatic increase in the use of polypropylene mesh. SUI treatment using mesh has, in some cases, led to severe and sometimes irreversible complications resulting in an FDA warning first released in 2008 and then again in 2011 [2, 3]. Subsequent to the warnings, there was a resurgence of interest in other forms of treatment for SUI [4]. Provided its limitations are resolved, UBT represents an ideal treatment for poor surgical candidates, patients with comorbidities, placing them in a high-risk category for anesthesia, as well as patients who opt against a more complex surgical procedure [5].

Complications related to the use of synthetic material in the treatment of SUI highlight a need for preclinical studies. Experimental animal models could be used to help assess and solve the fundamental problem associated with UBT, the absence of an ideal bulking material. The urethral bulking agent should be stable, easy to inject, nonimmunogenic, permanent, nonmigratory, nonerosive, and easily stored and handled and should possess a high safety profile [6]. All these properties can be tested on laboratory animals; however only a few such preclinical studies have been performed to date [7, 8]. We recently described a rat model for preclinical testing of urethral bulking agents, including hard microparticle bulking therapy [9]. In this study, we present a preclinical experiment comparing two new bioactive ceramic bulking materials to the three bulking agents most commonly used in treatment of SUI.

The two new bulking materials we tested were silica-calcium phosphate (SCPC10) and Cristobalite. Both of these bioactive ceramic particles have been studied extensively in bone regeneration [10]. Previous studies documented that bioceramics stimulate tissue attachment to the porous surface through serum protein adsorption, tissue formation, and cell attachment [11]. The chemical stability of bioceramics depends on the chemical composition and crystalline phases, which can be controlled by thermal treatment and compact pressure during the manufacturing process [12]. In addition, we used a new formulation of hyaluronic acid sodium salt, sodium hyaluronate, Hyaluronan (HA), with optimized viscosity, which gets absorbed by the body, leaving only the particles at the injection site where they can interact directly with the tissue. We suspended the two different experimental, bioceramic particles in HA and injected them into the proximal urethra of a rat. As a control, we used bulking materials that are currently in clinical use: silicon particles (Macroplastique, Uroplasty Inc, Minneapolis, MN, USA), calcium-hydroxyapatite particles (Copatite, Boston Scientific, Natic, MA, USA), and polyacrylamide hydrogel (Bulkamid, Contura International, Soeborg, Denmark). We used hard tissue histology techniques to evaluate particle integrity, incorporation, and host tissue reaction. In addition, we harvested the distal organs and tested them for the presence of particle and/or trace levels of their components.

## 2. Material and Methods

An individual bulking agent was injected into the proximal urethra of 21 female Wistar rats. Six animals per experimental group were injected with either SCPC10 or Cristobalite and three animals per control group were injected with Coaptite, Bulkamid, or Macroplastique. Organs from three healthy animals were harvested as normal controls.

### 2.1. Preparation of Bioceramic Particles, HA Suspension

HA, prepared in double-distilled water (ddH_2_O), was used as the suspension media for the two experimental bulking agents. According to our previous research, we used HA (molecular weight of 900 kDa) at a 2.5% concentration with a bioceramic particle to HA ratio of 1 : 4 [9]. The suspension mixture was sterilized in an autoclave for 20 minutes at 121°C. Viscosity of the 2.5% HA solution was approximately 80,000 cP prior to sterilization and decreased to approximately 30,000 cP following sterilization due to a slightly lower molecular weight, which is caused by heat-induced depolymerization of the HA molecule. pH of the material was 7.2 and remained within the physiological range after dissolving in water. Once sterilized, the solution was mixed on a mixing plate for 12 hours, or until the bioceramic particles were evenly distributed throughout the suspension media.

### 2.2. Injection

Female Wistar rats, 6-7 months old (Charles River Laboratories, Saint-Constant, Quebec, Canada), were maintained under standard laboratory conditions with free access to food and water. The University of Vermont Institutional Animal Care and Use Committee approved all animal procedures, and appropriate measures were taken to minimize pain and discomfort to the animals. Under inhalation anesthesia, through a lower midline abdominal incision, the mid urethra was bluntly dissected to expose the injection site. Using direct visualization with an operating microscope, the urethral bulking agent was injected into the wall of the mid urethra. This injection technique was previously described in depth [9]. Microphotographs were taken to record any gross abnormalities.

### 2.3. Tissue Harvesting

Five months after injection, under general inhalation anesthesia, a midline abdominal incision was made and the urethra was exposed. Following removal of the urethra, the lungs, kidneys, liver, and spleen were harvested to test for levels of particle ions. All tissues were immediately fixed in an ethanol-based fixative, HistoChoice (Amresco, Solon, OH), for 7 days at room temperature.

### 2.4. Histological Analysis

Urethral specimens were processed using a novel technique, which facilitates immunohistochemical analysis on tissues containing hard particles [13–15]. It involves embedding the specimen in resin and cutting the sections using a sawing microtome (Leitz 1600, Wetzlar, Germany). This method was previously described in detail [9]. Immunohistochemical staining was performed using a primary mouse monoclonal antibody specific to tumor necrosis factor-alpha (TNF-*α*, Abcam Ltd., Cambridge, UK). Additional sections were stained using Giemsa surface stain [16]. A pathologist evaluated the urethral cross-sections to determine bead morphology and host tissue reaction.

### 2.5. Scanning Electron Microscopy (SEM)

Bioceramic particles were analyzed with SEM prior to suspension and injection. The size, shape, and integrity of the bulking material were evaluated.

### 2.6. Evaluation of Distant Organs for the Presence of Particles and Their Components

Following tissue digestion, filtration, and centrifugation, the liver, kidneys, spleen, and lungs were examined microscopically for the presence of whole particles. Subsequently, organs were digested in 70% HNO_3_ and the concentration of Si, P, Ca, and Na was measured using inductively coupled plasma optical emission spectroscopy. A control experiment was run in parallel using organs from normal, noninjected animals.

### 2.7. Statistical Analysis

Quantitative values were presented as means ± standard error of the mean. All statistical calculations were performed using GraphPad PRISM (San Diego, CA, USA). Paired data were evaluated using a two-tailed Student's* t*-test. A one-way analysis of variance was used for comparisons of multiple groups.* P* values ≤ 0.05 were considered statistically significant.

## 3. Results

### 3.1. SEM Evaluation of Bioceramic Particles

**Figure 1:**
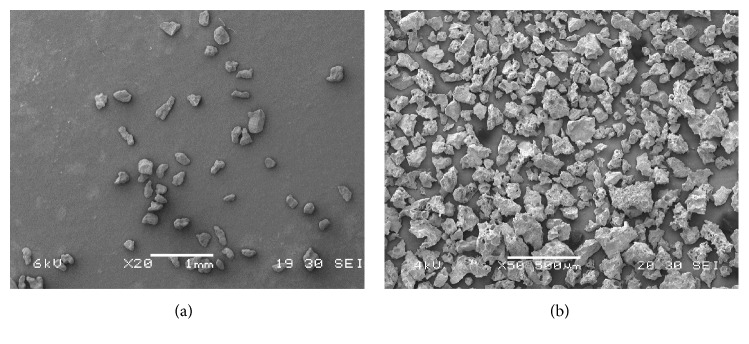
Scanning electron microscopy depicting the surface of (a) Cristobalite and (b) SCPC10 particles.

Cristobalite particles were cuboidal in shape, with a cross-section size of 75–200 micrometers. Significant variation in the shape and size was noted among the SCPC10 particles, which displayed a coral-like appearance with multiple pores (Figure 1).

### 3.2. Intraoperative Examination

In vivo examination under the operating microscope of Cristobalite and SCPC10 particles 5 month after injection did not reveal any gross abnormalities. No signs of abscess formation, acute inflammatory changes, or florid scarring of the host tissue were noted. These observations were comparable to those obtained immediately following injection.

### 3.3. Histological Examination

#### 3.3.1. SCPC10

**Figure 2:**
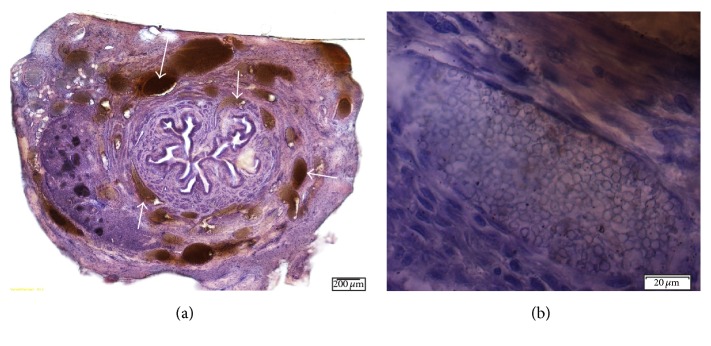
Cross-sections of urethra 5 months after injection of SCPC10 particles. (a) Cross-sectional view of entire urethra with nodular particle packets in the submucosa and adventitia (10x magnification). Arrows indicate particle masses. (b) Particle packet in a well circumscribed nodule showing some surface irregularities and size variability of the individual particles (20x magnification).

Cross-sections of the injection site showed coaptation of the urethral mucosa, with the particles located in the submucosa and urethral smooth muscle. Surface irregularities of individual particles were noted on high-power light microscopy. Collagen fibers were densely packed around the particle mass. Immunostaining did not reveal active macrophages, signs of a robust foreign body response, or scarring. Fibroblastic proliferation around individual particles resulted in a 1.5–2 times larger volume than the injected particle mass alone (Figure 2).

#### 3.3.2. Cristobalite

**Figure 3:**
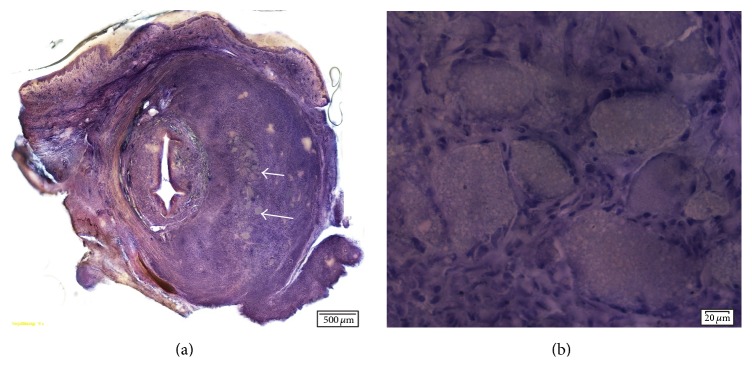
Cross-sections of rat urethra 5 months after injection of Cristobalite particles. (a) Cross-sectional view of the entire urethra (10x magnification). Arrows indicating particle mass. (b) Image showing particles with a slight variability in size and surrounding fibroblastic proliferation (20x magnification).

Particle aggregates were seen scattered throughout the urethral cross-sections. The aggregates were polyhedral in shape and were integrated into the tissue due to the surrounding mild host reaction. Fibroblastic proliferation around the particle masses and between the individual particles was noted. This proliferation resulted in a volume 2-3 times larger than the injected particle mass alone, which was noted on the urethral lumen. Some histiocytes were interspersed among the fibroblasts, indicating a mild histiocytic reaction. The particles were seen as a mass in the adventitia as well as the submucosa of the urethra (Figure 3). Overall, for both SCPC10 and Cristobalite, a minor host tissue response without florid scarring was visualized using light microscopy. The presence of macrophages and a host tissue inflammatory response was ruled out with negative TNF alpha staining.

#### 3.3.3. Coaptite

**Figure 4:**
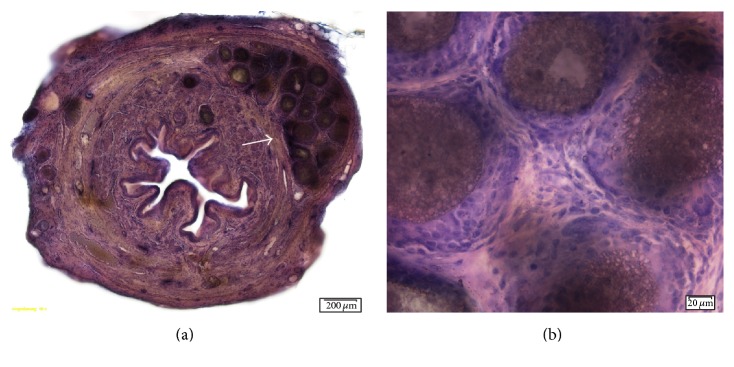
(a) Cross-sections of urethra 5 months after injection of Coaptite (10x magnification). Arrow pointing to particle mass. (b) Image showing particles surrounded by fibroblasts and giant cells. Relatively uniform size of the injected particles.

Compared to the bioceramic particles, there were less fibroblastic changes with less mass effect. The increase in volume was approximately twofold. Fibroblastic proliferation was more localized and nodular and was surrounded by giant cells. The injected material was primarily seen in aggregates, with each aggregate containing a variable amount of material. There were also aggregates of histiocytes with giant cells interspersed. The contour of each individual calcium hydroxylapatite particle was relatively uniform. Coaptite showed calcium-hydroxyapatite particles uniform in size (70–125 microns) surrounded by fibroblasts and collagen (Figure 4).

#### 3.3.4. Bulkamid

**Figure 5:**
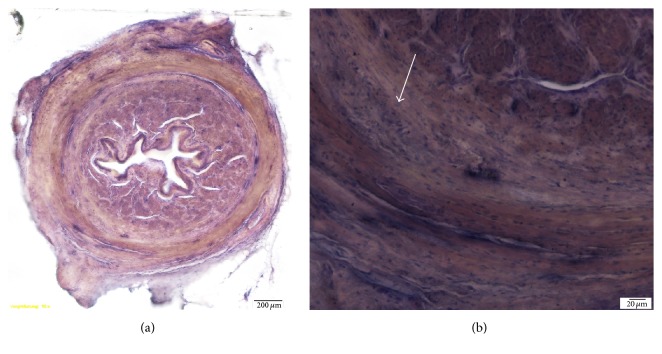
Cross-sections of urethra 5 months after injection of Bulkamid. (a) Cross-section of urethra without large injection mass (10x magnification). (b) Image showing small injection aggregates in a nodular type of reaction with mild surrounding fibroblastic changes (20x magnification). Arrow indicates bulking agent mass.

There were two distinct types of host tissue reaction: diffuse fibroblastic changes and nodular aggregates with mild fibroblastic proliferation. The diffuse changes were surrounded by fibroblasts, resulting in an increased volume of approximately 3-fold. The nodular type of reaction resulted in a volume increase of approximately 2-fold. The volume effect and fibroblastic reaction seen in the nodular tissue changes were similar to those seen with Coaptite. There was a large variation in size of the nodular aggregates spread throughout the tissue. Distribution of the bulking agent was seen in the adventitia and submucosa. The only tissue change noted with Bulkamid was mild fibroblastic proliferation (Figure 5).

#### 3.3.5. Macroplastique

**Figure 6:**
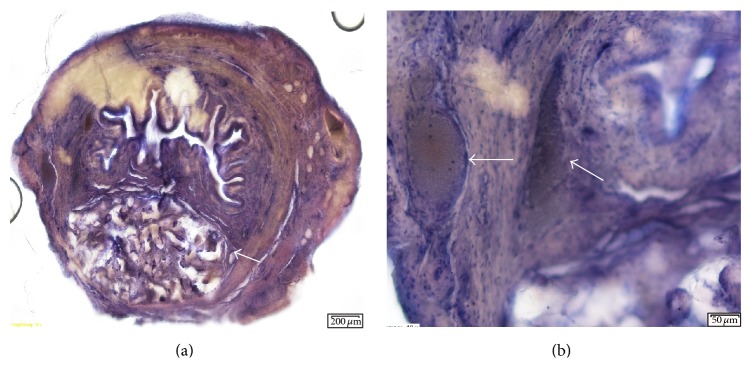
Cross-sections of urethra 5 months after injection of Macroplastique. (a) Cross-section of entire urethra (10x magnification). Arrows indicate bulking material mass in the adventitia and suburothelium. (b) Injected bulking material with some surrounding fibroblastic proliferation (20x magnification).

This bulking agent also displayed two distinct reaction types to the injected material: nodular aggregates and a more diffuse fibroblastic type of reaction. The nodular aggregates of material appeared to be somewhat band-like, partially folded onto itself, and surrounded predominantly by histiocytes. This resulted in an increase in volume of approximately 1.5-fold. The more diffuse reaction is primarily a fibroblastic reaction with mild, thin proliferation surrounding the injected bead masses. The injected material aggregates varied significantly in size and shape. Individual silicone beads varied in size by a factor of 8. Particles were organized in collagen-encapsulated clusters with evidence of mild fibroblast in-growth (Figure 6).

### 3.4. Evaluation of Distant Organs

**Figure 7:**
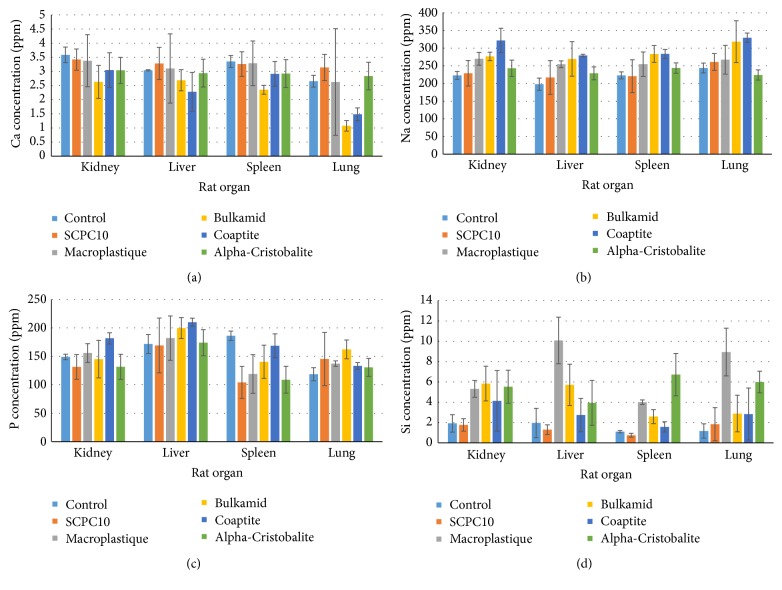
Bar graphs summarizing the ion levels of particle components in the distant organs.

There were no particles detected in the distant organs. Animals injected with Cristobalite and Macroplastique appeared to have higher Si concentrations in the lungs; nevertheless, the ion concentrations in all of the organs were within the biological range. Trace components (Si, P, Ca, and Na elements) from SCPC10 and Cristobalite showed comparable concentrations to the other injected bulking agents and the healthy, intact controls (Figure 7).

## 4. Discussion

The use of urethral bulking therapy declined significantly over the course of the last two decades, in part, due to an increase in the use of anti-incontinence procedures using polypropylene mesh. It has been well documented that this therapy is not suitable for every patient and can be associated with serious complications [17, 18]. Complications from UBT are linked to the degradation of synthetic material, causing local inflammatory changes, or distant migration of the material or its components [19, 20]. UBT-induced complications were most pronounced with the use of polytetrafluoroethylene (Telfon), popularized in the 1970s [21]. Teflon is not a biologically inert substance, which leads to significant scarring as well as a foreign body granulomatous reaction locally and distantly from migration of the particles [22, 23].

We compared two types of bioactive ceramic particles, suspended in sodium hyaluronate, to bulking materials currently being used clinically, and assessed the propensity of these materials to cause complications due to local host tissue reaction and distant migration. Sodium hyaluronate is the sodium salt of hyaluronic acid. It is a natural, linear, unbranched polysaccharide belonging to the class of nonsulphated glycosaminoglycans. It occurs naturally in the human body, especially in the vitreous humor and synovial fluid, and as a major component in the extracellular matrix in the skin. It proved to be the optimal suspension media. Its high viscosity prevents plugging of the needle and facilitates uniform particle suspension. The propensity for absorption allows the surface of the bioceramic particles to better interact with the host tissue, preventing migration and providing an optimal bulking mass. The rate at which Hyaluronan gets absorbed by the body is tissue specific and depends on the flow of body fluids in the specific tissue as well as the viscosity. An additional advantage is that it does not significantly change its properties during the sterilization process [24].

Injections of SCPC10 particles showed fibroblastic proliferation, which is needed to hold the particle mass in place. Significant variation in particle size raises the concern that they may migrate to distant organs, which was previously documented on particles smaller than 80 microns [25]. However, no evidence of migration of particles or their components was documented in this study. When compared to the commonly used bulking agents, both SCPC10 and Cristobalite produced a more robust fibroblastic and histiocytic reaction, which in turn helped to integrate and encapsulate the particle aggregates. In vivo, this property effectively anchors the particles in the target tissue and generates a larger bulking effect. Cristobalite, which has the same inherent properties as SCPC10 but shows more regularity in shape and size, induced stronger fibroblastic proliferation, which in turn resulted in a larger bulking effect. Microscopic examination of tissue injected with Cristobalite was most similar to the response seen with Macroplastique but displayed some important differences. Macroplastique particle aggregates were encapsulated by a thin fibroblastic and histiocytic reaction causing less of a mass effect. When comparing all particle types, it appears that round particles elicit less of a fibroblastic tissue reaction.

Comparable levels of Ca, Na, and P ions were noted in the distant organs of all experimental animals. Animals injected with SCPC10 had comparable Si levels to healthy, noninjected controls and animals injected with Cristobalite and Macroplastique appeared to have higher concentrations of Si than controls. Nevertheless, the concentration in all organs was within the biological range [26]. The particle migration seen with previously introduced bulking agents represents a major complication, which has serious and even fatal consequences [27]. For some substances (Teflon, Durasphere, and autologous fat), migration was detected only after broader clinical use. Testing for the presence of particle ions is a novel method that provides additional evidence on the safety of these new materials. The absence of particles and their components in distant organs, shown in our study, provides initial safety data. However, experiments conducted using a rodent model do not provide a final guarantee of clinical safety. We plan to repeat these preclinical trials using larger animals.

In this study, we performed a comprehensive, side-by-side comparison of urethral bulking agents utilizing a previously established rat animal model in combination with hard tissue histologic and immunohistochemical analysis. On this basis, two newly developed bioactive ceramic particles, suspended in sodium hyaluronate, were compared to three currently used urethral bulking agents. The local host tissue response and bulking effects of both SCPC10 and Cristobalite were superior while also possessing a comparable safety profile. Although further studies are necessary, this research suggests that Cristobalite, suspended in sodium hyaluronate, produces a superior bulking effect and can provide the improved, durable long-term response needed for successful urethral bulking therapy.
